# Modelling BMI Trajectories in Children for Genetic Association Studies

**DOI:** 10.1371/journal.pone.0053897

**Published:** 2013-01-17

**Authors:** Nicole M. Warrington, Yan Yan Wu, Craig E. Pennell, Julie A. Marsh, Lawrence J. Beilin, Lyle J. Palmer, Stephen J. Lye, Laurent Briollais

**Affiliations:** 1 School of Women’s and Infants’ Health, The University of Western Australia, Perth, Western Australia, Australia; 2 Samuel Lunenfeld Research Institute, Mount Sinai Hospital, Toronto, Ontario, Canada; 3 School of Medicine and Pharmacology, The University of Western Australia, Perth, Western Australia, Australia; 4 Ontario Institute for Cancer Research, Toronto, Ontario, Canada; John Hopkins Bloomerg School of Public Health, United States of America

## Abstract

**Background:**

The timing of associations between common genetic variants and changes in growth patterns over childhood may provide insight into the development of obesity in later life. To address this question, it is important to define appropriate statistical models to allow for the detection of genetic effects influencing longitudinal childhood growth.

**Methods and Results:**

Children from The Western Australian Pregnancy Cohort (Raine; n = 1,506) Study were genotyped at 17 genetic loci shown to be associated with childhood obesity (FTO, MC4R, TMEM18, GNPDA2, KCTD15, NEGR1, BDNF, ETV5, SEC16B, LYPLAL1, TFAP2B, MTCH2, BCDIN3D, NRXN3, SH2B1, MRSA) and an obesity-risk-allele-score was calculated as the total number of ‘risk alleles’ possessed by each individual. To determine the statistical method that fits these data and has the ability to detect genetic differences in BMI growth profile, four methods were investigated: linear mixed effects model, linear mixed effects model with skew-*t* random errors, semi-parametric linear mixed models and a non-linear mixed effects model. Of the four methods, the semi-parametric linear mixed model method was the most efficient for modelling childhood growth to detect modest genetic effects in this cohort. Using this method, three of the 17 loci were significantly associated with BMI intercept or trajectory in females and four in males. Additionally, the obesity-risk-allele score was associated with increased average BMI (female: β = 0.0049, P = 0.0181; male: β = 0.0071, P = 0.0001) and rate of growth (female: β = 0.0012, P = 0.0006; male: β = 0.0008, P = 0.0068) throughout childhood.

**Conclusions:**

Using statistical models appropriate to detect genetic variants, variations in adult obesity genes were associated with childhood growth. There were also differences between males and females. This study provides evidence of genetic effects that may identify individuals early in life that are more likely to rapidly increase their BMI through childhood, which provides some insight into the biology of childhood growth.

## Introduction

Obesity is a major global public health problem. The World Health Organisation estimated in 2010 there were at least 42 million overweight children under the age of 5-years and one billion overweight adults globally [1]. Childhood obesity is associated with poor mental [2], [3], [4], [5] and physical health [6], [7] and is one of the strongest predictors of adult obesity [8], [9]. Adult obesity, in turn, increases the risk of many diseases including coronary heart disease, metabolic syndrome, some cancers, stroke, liver and gallbladder disease, sleep apnoea and respiratory problems, osteoarthritis and gynaecological problems [1]. It has been proposed that there are critical periods early in an individual’s life for the development of obesity including gestation and early infancy, adiposity rebound and adolescence [10].

An individual’s susceptibility to obesity is thought to result from a combination of their genetics, behaviours and environment. The heritability of obesity is estimated from family and twin studies to be between 40 and 80% [11], [12], [13], which appears to be age dependent with younger individuals having higher heritability estimates [14]. Genetic factors have an important role in childhood obesity, but their role may be different to those that operate in adulthood. Since the advent of genome-wide association studies (GWAS), common variants within 35 genes have been discovered to be associated with adult obesity [15], [16], [17], [18], [19] and a further 48 genes associated with population variation in body mass index (BMI) and weight [20], [21], [22], [23], [24], [25], [26] in individuals of European descent. In particular, common variants within the fat-mass and obesity associated (FTO) and melanocoritin 4 receptor (MC4R) genes are associated with modest effects on BMI (0.2–0.4 kg/m^2^ per allele) which translate into increased odds of obesity of 1.1–1.3 in adults [24], [26], [27], [28], [29]. However, the genomic regions discovered to date to be associated with BMI account for less than 1% of the total variance in the BMI [30], leaving much of the estimated heritability unexplained. In addition, relatively few studies have investigated the association between the adult BMI associated variants and childhood BMI [23], [31], [32], [33], [34]. Zhao et al [31] investigated the association between childhood BMI and 13 genomic loci reported to be associated with adult obesity to find that nine of the loci contribute to paediatric BMI between birth and 18 years of age. Subsequently, several authors have investigated the association between adult BMI loci and changes in growth over childhood. Hardy and colleagues [33] took variants from the two most commonly reported obesity genes, FTO and MC4R, to see if they were associated with life course body size. They found the association with BMI in both genes strengthened during childhood up until 20 years of age before weakening throughout adulthood. In 2010, Elks et al [34] used eight variants that showed individual associations with childhood BMI to create an obesity-risk-allele-score. This allele-score was strongly associated with early infant weight gain but also with weight gain over childhood. Finally, den Hoed et al [32] looked at BMI in childhood and adolescence against a larger subset of replicated SNPs representing the 16 BMI loci from the six genome-wide association studies in adults of white European descent [22], [23], [24], [26], [35], [36]. Together, these studies begin to provide evidence that genetic loci associated with BMI in adulthood start having an effect in childhood and even infancy.

Obesity develops over a period of time so investigating the genetic determinants underlying this developmental process may provide insights into mechanisms of the genetic associations. Sophisticated longitudinal analyses allow questions to be addressed that cannot be determined from cross-sectional analyses. These longitudinal models assess patterns and duration of genetic effect at baseline and over a time period and the differences in means and rates of change of a trait. It is therefore important to investigate the genetic component of BMI trajectory in order to better understand some of the underlying biology of growth. The analysis of longitudinal growth curves allows one to identify specific stages in which genes play a central role.

A child’s growth rate profile often contains important information regarding their genetic make-up and environmental exposures; however, BMI trajectories are difficult to model statistically due to the various changes in growth rate over childhood. Children tend to have rapidly increasing BMI from birth to approximately 9 months of age where they reach their adiposity peak; BMI then decreases until around the age of 5–6 years at adiposity rebound and then steadily increases again until after puberty where it tends to plateau through adulthood. These patterns of growth tend to be different in males and females where females often reach each of the ‘landmarks’ (adiposity rebound, puberty and plateau at adult BMI) at an earlier age than males. These changes over time within each individual, as well as the increasing variability over time of BMI between individuals, are often difficult to capture accurately in a statistical model. This is particularly the case when the aim is to detect modest genetic effects. The World Health Organization recently conducted research into statistical methods used to estimate growth curves over childhood and examined 30 previously published methods, of which only 7 could handle multiple measurements per child [37]. These methods range from non-linear, parametric curves [38] to non-linear, non-parametric methods where the form of the curve was allowed to differ for each subject [39], [40] and from linear mixed-effects models for longitudinal normally distributed data [41], [42] to a more general multilevel model, some with non-parametric components [43], [44], [45]. Although many methods have been previously used for growth modelling, not all are appropriate for genetic association analyses or modelling growth profiles in longitudinal birth cohorts.

We aim to compare various modelling approaches to assess the genetic effects of BMI growth through infancy, childhood and adolescence. To investigate the sensitivity of these different modelling frameworks to detect genetic effects, we will use the previously published adult obesity and BMI associated SNPs that have been shown to be associated with childhood BMI and explore their associations with childhood growth.

## Methods

### Subjects

The Western Australian Pregnancy Cohort (Raine) Study [46], [47], [48] is a prospective pregnancy cohort where 2,900 mothers were recruited prior to 18-weeks’ gestation between 1989 and 1991. Recruitment took place at Western Australia’s major perinatal centre, King Edward Memorial Hospital, and nearby private practices. The mothers completed questionnaires regarding the children and the children had physical examinations at average ages of 1, 2, 3, 6, 8, 10, 14 and 17 years. A DNA sample was collected at the 14 and 17 year follow-ups. A subset of 1,506 individuals were used for analysis in this study using the following inclusion criteria: at least one parent of European descent, live birth, unrelated to anyone in the sample (one of every related pair, including multiple births, was selected at random to exclude), no significant congenital anomalies, a DNA sample and at least one measure of body mass index (BMI) throughout childhood. Weight and height were measured at each follow-up by trained members of the research team [49]; weight was measured using a Wedderburn Digital Chair Scale to the nearest 100 g with children dressed in running shorts and a singlet top and height was measured to the nearest 0.1 cm with a Holtain Stadiometer. BMI was calculated from the weight and height measurements (median 6 measures per person, interquartile range 5–7, range 1–8 measurements), with a total of 8,986 BMI measures. The study was conducted with appropriate institutional ethics approval from the King Edward Memorial Hospital and Princess Margaret Hospital for Children ethics boards, and written informed consent was obtained from all mothers. The cohort has been shown to be representative of the population presenting to the antenatal tertiary referral centre in Western Australia [48].

### Genes

We wanted to investigate markers that have an effect on childhood BMI, and more importantly, change in BMI over childhood so selected the 17 genetic variants published in den Hoed et al [32]. These SNPs were first discovered to be associated with adult BMI and replicated in at least one study against childhood BMI and change in BMI growth over childhood. At the time of selecting SNPs for this study, they were the largest set of SNPs shown to be associated with BMI over childhood and adolescence. We did not include loci that have been shown to be associated with only obesity risk but not BMI. Subsets of these 17 SNPs (either the same SNPs or a SNP in high LD [r^2^>0.8]) were also presented by Elks et al [34] and Hardy et al [33], who showed associations with changes in growth over childhood. Genetic information on these 17 published genetic variants was available for individuals in our sample, either directly genotyped SNPs (rs925946 (BDNF), rs10913469 (SEC16B), rs2605100 (LYPLAL1), rs987237 (TFAP2B), rs10838738 (MTCH2), rs7138803 (BCDIN3D) and rs10146997 (NRXN3)) or from the best guess genotype data imputed against HapMap release 22 (rs2815752 (NEGR1), rs6548238 (TMEM18), rs7647305 (ETV5), rs10938397 (GNPDA2), rs613080 (MRSA), rs1488830 (BDNF), rs8055138 (SH2B1), rs1121980 (FTO), rs17782313 (MC4R) and rs11084753 (KCTD15)). Genotyping and quality control has been described elsewhere [50]. Briefly, our sample was genotyped using the genome-wide Illumina 660 Quad Array. Genotyping was performed on the Illumina BeadArray Reader at the Centre for Applied Genomics, Toronto, Canada using 250 nanograms of DNA. The genotype data was cleaned using standard thresholds (HWE p-value >5.7×10^−7^, call rate >95% and minor allele frequency >1%). Individual level genotype data was extracted for those SNPs of interest that were directly genotyped by the chip and passed QC measures. Imputation of un-typed or missing genotypes was also performed using MACH v1.0.16 for the all 22 autosomes with the CEU samples from HapMap Phase2 (Build 36, release 22) used as a reference panel. Two variants in the BDNF gene were investigated as they have previously been shown to be independently associated with obesity [22] (r^2^ = 0.11). The 17 SNPs are described in Table S1, including the available sample size with complete data for each SNP. These 17 SNPs were used to investigate the sensitivity of each method to detect genetic variants in terms of point estimates and standard errors (SEs) across various time points (for those methods that could be compared). Each SNP was incorporated into the model independently assuming an additive genetic effect for the obesity risk allele. In addition, an ‘obesity-risk-allele score’ was created on the subset of individuals with complete genetic data by summing the number of risk alleles an individual had (n = 1,219) [51]. The alleles were not weighted by their effect size as this has previously been shown to only have limited benefit [52].

### Statistical Analysis

Four popular methods were compared to assess the accuracy of estimation of BMI growth trajectories and the ability to detect genetic effects influencing these trajectories. These methods included: Linear Mixed Effects Model (LMM) [41], the Skew-*t* Linear Mixed Effects Model (STLMM) [53], [54], [55], Semi-Parametric Linear Mixed Models (SPLMM) and a Non-Linear Mixed Model (NLMM), also known as SuperImposition by Translation and Rotation (SITAR) [40]. Although there are many possible statistical methods that could be utilized in this context, these methods were chosen as they allow for adjustment of potential confounders, appropriately account for the complex correlation structure between the repeated measures, allow for incomplete data on the assumption that data are missing at random, and are computationally feasible in the context of candidate gene and genome-wide association studies. Once the best fitting model was defined for each method, the model fit for each of the methods was compared. A small simulation study was also conducted using re-sampling techniques based on 1,000 non-parametric bootstrap data sets with replacement [56] from the Raine data and calculating an R^2^ statistic for each method fit to these simulated datasets.

#### LMM

The LMM with a polynomial function is a common tool for growth curve analysis with continuous repeated measures. For a set of time points varying from *1*,.,*t*, the time trend in the sample can be described by a (*q*-1)st-degree polynomial function, with q ≤ t. The growth curve LMM for the j^th^ individual and t*^th^* time point and with the time scale measured by age is as follows:
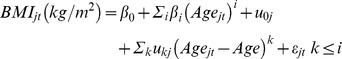
Where Age is the mean age over the *t* time points in the sample (i.e. 8 years), β_i_ are the parameter estimates for the fixed effects, u_kj_ are the parameter estimates for the random effects assumed multivariate normal and the ε_jt_‘s are the error terms assumed normally distributed *N*(0, *Σ*), where *Σ* is the within-individual correlation matrix. Both age and the natural log transformation of age were considered as the time component to identify the optimal underlying scale. Both fixed (*i*) and random (*k*) effects up to polynomial of degree 3 were tested for significance. Several within-individual correlation structures were considered, including autoregressive, continuous autoregressive, exchangeable (compound symmetric) and unstructured.

Following the guidelines outlined in Cheng et al [57], the initial saturated model considered included a cubic function of age for both the fixed and random effects and BMI on the natural log scale, was used to compare covariance (random effects) matrices. Initially, likelihood ratio tests (LRT) were used to assess the required degree of polynomial function for the random effects to fit the data accurately, while keeping the fixed effects the same and specifying an independence correlation matrix for the random effects. Next, a similar approach was used to investigate within-individual correlation structures in addition to the random effects. Finally, models with both untransformed and natural log transformed age were compared using diagnostic plots such as fitted verses observed values, fitted versus residual values and distribution of both random effects and error terms.

#### STLMM

The assumption of multivariate normal random effects and within-subject errors is often violated, particularly when modelling the childhood growth curve. This may lead to biased estimation of fixed effects and their SEs and thus to wrong statistical inference, in particular of the genetic association-related parameters. A common approach to achieve normality is to transform the response variable but generally there is not a unique transformation that could be used and the results of the analyses might depend on the transformation used. To avoid transforming the response and still obtain a valid inference under a non-normal distribution assumption for the response, we utilised an extension of the LMM model assuming a multivariate *t* distribution for the error terms, ε_jt_‘s, and a multivariate skew-normal distribution for the random effects. The resulting model for the response over the *t* time points is multivariate skew-*t* with specific parameters that account for the asymmetry (skewness parameters) and long-tail (degree of freedom of the t distribution) of the response distribution [54]. The specification in terms of fixed and random effects was identical to the LMM. No transformations were applied to either BMI or age as the skewness in the data was accounted for by the model structure.

#### SPLMM

Semi-parametric linear mixed models make use of smoothing splines, which yield a smoother growth curve estimate than the polynomial function in the LMM when fitting non-linear relationships. The basic model for the j^th^ individual and time-point *t* is as follows:
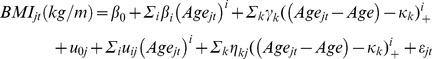



****Where κ_k_ is the *k*-th knot and (t – κ_k_)_+_ = 0 if t ≤ κ_k_ and (t – κ_k_) if t>κ_k_, which is known as the truncated power basis that ensures smooth continuity between the time windows.

Various numbers and positions of knots and the degree of polynomial between knots were compared to find the best fit to the data. Knot points were initially estimated visually from both individual profiles and the population average curve in males and females separately. To optimise the number and placement of the knot points, we fit a series of models with the knot points placed at 6-month intervals around the estimated knot points and incorporated additional knot points to see if they improved the model fit. The model with the lowest Akaike Information Criterion (AIC) was selected as the final model. Finally, we investigated the degree of polynomial, up to the third degree, required for each spline, once again selecting the best model with the lowest AIC.

#### NLMM

The SITAR method [40] was recently defined to summarize height growth in puberty (in particular peak height velocity) and estimate subject-specific parameters that can be used to investigate relationships with earlier exposures and later outcomes. The SITAR method (referred to here as NLMM) model has a single fitted curve at the population level and individual level estimates of mean differences in size (shifting up or down of the BMI curve), growth tempo (left-right shift of the curve on the age scale) and velocity (shrinking or stretching of the age scale).

The basic model for the growth curves is:
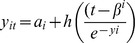
Where:

*y_it_* = growth of subject *i* at age *t.*

*h(t)* = natural cubic spline curve of growth vs. age.

*α_i_* = random growth intercept that adjusts for differences in mean height (*size*).

*β_i_* = random growth intercept to adjust for difference in timing (*tempo*).

*γ_i_* = random age scaling adjusting for the duration of the growth spurt (*velocity*).

This model was fit with the three parameters (size, tempo and velocity) as random effects, size and velocity as fixed effects, and h(t) a natural cubic spline curve with 3 to 8 degrees of freedom (df) fitted as fixed effects. BMI and age were fitted both untransformed and natural log transformed, to identify the best fit to the data. Model fit to the data were compared using AIC, deviance and residual standard deviation. The estimates for the three parameters (size, tempo and velocity) were extracted for each individual and used for genetic analyses.

Given that growth curves differ greatly between males and females, particularly around puberty, and because different genes may influence the timing of growth spurts in males and females, sex stratified models were used for all analyses. Age was mean centred prior to analysis. Due to the possibility of population stratification in our sample given our sampling criteria of at least one parent of European descent, a sensitivity analysis was conducted adjusting the genetic analyses for the first five principal components generated in the EIGENSTRAT software [58]. No adjustment for multiple testing have been made as our goal was to estimate a combined effect of SNPs that have already been validated in previous studies and shown to be significantly associated with childhood BMI and growth. All analyses were conducted in *R* version 2.12.1 [59]; the spida library was used for the SPLMM models and the sitarlib library was used for the NLMM models. To enable comparison between the four methods, maximum likelihood estimation was used for all mixed models. Genetic loci were considered associated with BMI if the global likelihood ratio test was significant at a α<0.05 level.

## Results

### Population Characteristics

Of the 1,506 children in the analysis, there are 773 males (51%) and 733 females. Table 1 gives the characteristics of the Raine sample used in the analysis. At birth, these babies were similar to the Western Australian population of births with an average birth weight of 3.35 Kg (SD = 0.59 Kg) and gestational age of 39.35 weeks (SD = 2.11 weeks), 25.21% of them were born to mothers who smoked throughout pregnancy and 8.77% born preterm. The mothers on average gained 8.79 kg (SD = 3.78) throughout pregnancy and breast fed their infant for an average of 6 months (IQR = 2–12 months). On average, the infants gained 6.98 Kg (SD = 1.17 Kg) in the first year of life.

**Table 1:** The phenotypic characteristics of the Raine sample.

		All	Male	Female	P-Value
		(n = 1,506)	(n = 773)	(n = 733)	
Age	Year 1 (n = 1,375)	1.16 (0.10)	1.15 (0.10)	1.16 (0.10)	0.22
(yr)	Year 2 (n = 402)	2.18 (0.14)	2.19 (0.14)	2.16 (0.14)	0.05
	Year 3 (n = 994)	3.11 (0.12)	3.12 (0.13)	3.11 (0.10)	0.71
	Year 5 (n = 1,324)	5.92 (0.18)	5.91 (0.19)	5.92 (0.18)	0.30
	Year 8 (n = 1,320)	8.10 (0.35)	8.12 (0.34)	8.09 (0.36)	0.17
	Year 10 (n = 1,274)	10.60 (0.18)	10.60 (0.19)	10.59 (0.17)	0.16
	Year13/14 (n = 1,276)	14.07 (0.20)	14.07 (0.20)	14.07 (0.19)	0.55
	Year 16/17 (n = 1,021)	17.05 (0.25)	17.03 (0.24)	17.06 (0.25)	0.06
BMI	Year 1 (n = 1,375)	17.11 (1.40)	17.38 (1.38)	16.82 (1.37)	4.63E-14
(kg/m^2^)	Year 2 (n = 402)	15.97 (1.29)	16.19 (1.28)	15.72 (1.25)	2.00E-04
	Year 3 (n = 994)	16.15 (1.27)	16.29 (1.21)	16.00 (1.31)	2.00E-04
	Year 5 (n = 1,324)	15.86 (1.76)	15.88 (1.70)	15.84 (1.82)	0.64
	Year 8 (n = 1,320)	16.88 (2.54)	16.79 (2.47)	16.97 (2.62)	0.29
	Year 10 (n = 1,274)	18.69 (3.41)	18.58 (3.38)	18.80 (3.45)	0.25
	Year13/14 (n = 1,276)	21.45 (4.23)	21.21 (4.24)	21.71 (4.20)	0.03
	Year 16/17 (n = 1,021)	23.02 (4.38)	22.83 (4.34)	23.23 (4.42)	0.15
Height	Year 1 (n = 1,375)	0.78 (0.03)	0.78 (0.03)	0.77 (0.03)	1.04E-14
(m)	Year 2 (n = 402)	0.90 (0.03)	0.91 (0.03)	0.90 (0.03)	3.00E-04
	Year 3 (n = 994)	0.96 (0.04)	0.97 (0.04)	0.96 (0.04)	1.06E-09
	Year 5 (n = 1,324)	1.16 (0.05)	1.17 (0.05)	1.15 (0.04)	6.05E-07
	Year 8 (n = 1,320)	1.29 (0.06)	1.30 (0.06)	1.29 (0.06)	4.37E-06
	Year 10 (n = 1,274)	1.44 (0.06)	1.44 (0.07)	1.44 (0.06)	0.97
	Year13/14 (n = 1,276)	1.65 (0.08)	1.67 (0.09)	1.62 (0.06)	4.94E-26
	Year 16/17 (n = 1,021)	1.73 (0.09)	1.79 (0.07)	1.66 (0.06)	1.94E-143
Weight	Year 1 (n = 1,375)	10.34 (1.24)	10.67 (1.24)	9.99 (1.15)	5.03E-25
(kg)	Year 2 (n = 402)	13.03 (1.49)	13.39 (1.48)	12.65 (1.40)	3.37E-07
	Year 3 (n = 994)	15.06 (1.84)	15.42 (1.83)	14.69 (1.78)	3.99E-10
	Year 5 (n = 1,324)	21.48 (3.37)	21.75 (3.42)	21.20 (3.30)	2.91E-03
	Year 8 (n = 1,320)	28.42 (5.68)	28.58 (5.65)	28.24 (5.72)	0.28
	Year 10 (n = 1,274)	39.01 (9.02)	38.80 (9.09)	39.23 (8.95)	0.40
	Year13/14 (n = 1,276)	58.49 (13.44)	59.50 (14.49)	57.39 (12.11)	4.81E-03
	Year 16/17 (n = 1,021)	68.69 (14.59)	73.15 (14.91)	64.12 (12.74)	3.91E-24
Number of follow-ups per person		5.97 (1.52)	5.96 (1.52)	5.97 (1.53)	0.91
Birth Weight (kg)		3.35 (0.59)	3.41 (0.59)	3.28 (0.58)	3.85E-05
Gestational Age (wks)		39.35 (2.11)	39.37 (2.05)	39.32 (2.17)	0.66
Preterm [% (N)]		8.77% (132)	8.03% (62)	9.55% (70)	0.34
Maternal smoking during pregnancy [% (N)]		25.22% (379)	22.77% (176)	27.81% (203)	0.03

Continuous variables are expressed as means (SD); binary variables as percentage (number).

### Model Fitting and Comparisons

The optimal model for each method was defined before any cross-method comparisons were conducted. The selected models for each method are summarized in Table 2.

**Table 2:** Characteristics of the best model for each method.

		Scale of response	Fixed effect parameters	Random effect parameters	Within-individual correlation matrix
Female	LMM	ln(BMI)	1+ age+age^2^+age^3^	1+ age+age^2^	corCAR1
	STLMM	BMI	1+ age+age^2^+age^3^	1+age	None
	SPLMM	ln(BMI)	piecewise cubic spline function ofage with knots at 2, 8 and 12 years	1+ age +0.5*age^2^	None
	NLMM	ln(BMI)	size and a natural cubic spline function of ln(age) for velocity with 3df	size and a natural cubic spline functionof ln(age) for tempo and velocity parameters with 3df	corCAR1
Male	LMM	ln(BMI)	1+ age+age^2^+age^3^	1+ age+age^2^	corCAR1
	STLMM	BMI	1+ age+age^2^+age^3^	1+age	None
	SPLMM	ln(BMI)	piecewise cubic spline function ofage with knots at 2, 8 and 12 years	1+ age +0.5*age^2^	None
	NLMM	ln(BMI)	size and a natural cubic spline function of ln(age) for velocity with 4df	size and a natural cubic spline functionof ln(age) for tempo and velocity parameters with 4df	corCAR1

#### LMM

The optimal LMM model for both males and females was based on ln(BMI) and untransformed age, with cubic polynomial of age in the fixed effects, a quadratic polynomial of age in the random effects and a continuous autoregressive correlation structure of order one. Hence, the final model for both females and males was
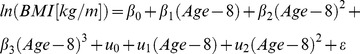



#### STLMM

The LMM model defined previously was used for this method; however BMI was modelled on the untransformed scale as the method accounts for the skewness and kurtosis of the BMI distribution. The model would not converge with both linear and quadratic age components in the random effects so this was reduced to only linear age. This was the most computationally intensive method to fit as it uses an expectation-maximization (EM) algorithm for parameter estimation, and hence took the longest time to converge.

#### SPLMM

For females, the optimal model had three knot points placed at two, eight and 12 years with a cubic slope for each spline. The males displayed a similar curve to the females, also with three knots at two, eight and 12 years and a cubic slope between each knot.

#### NLMM

The optimal model for females had a natural cubic spline curve with three degrees of freedom and both BMI and age on the natural log transformed scale. Similarly, the optimal model for males was with BMI and age on the natural log transformed scale but with four degrees of freedom for the natural cubic spline curve.

#### Comparisons

**Figure 1:**
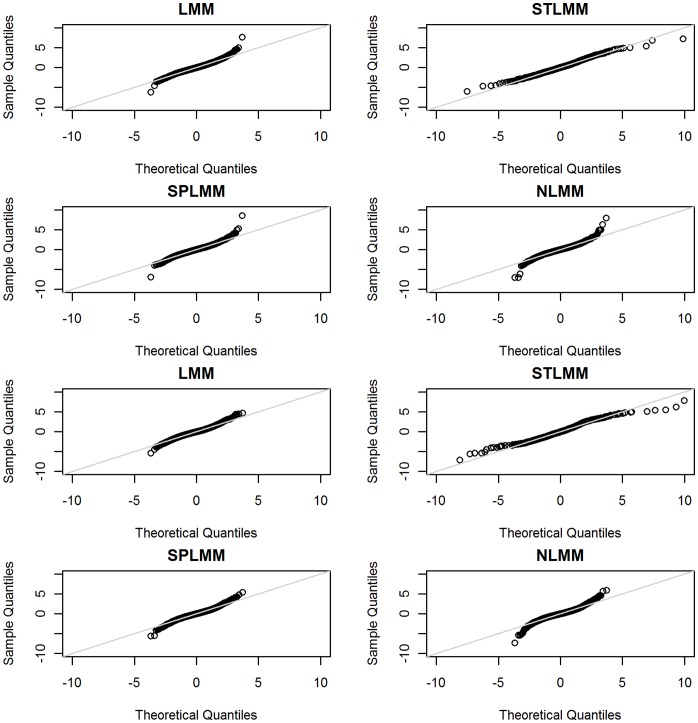
Q-Q plot of residuals for each of the methods by females (top four) and males (bottom four).

Table 3 displays the measures of fit used to compare methods: R^2^, R^2^ from 1,000 simulated datasets, observed-fitted values, number of SNPs detected and computational time. The R^2^, in conjunction with interquartile range of variation of R^2^ estimated through simulations, clearly favour the SPLMM as the best model fit for the females. The R^2^ estimates from the simulations indicate that although the STLMM method has higher R^2^ for both females and males, the interquartile range is much larger for STLMM method, indicating the model fit is more data dependent than the other methods, which is not desirable for generalization to other cohorts. The conclusion for the males is not as simplistic as the R^2^ is largest for the STLMM, however with the considerably longer computational time and the larger deviation the fitted values are from the observed values indicates that this model might not be appropriate for large scale genetic studies. Figure 1 displays the residuals from all four methods in both males and females. The female residual plots indicate the LMM, STLMM and SPLMM methods all have residuals distributed close to the expected distribution (normal for the LMM and SPLMM and skew-*t* for the STLMM). Several within-subject outliers (at the tails of the distribution) were not captured in all methods. However, the NLMM in particular had additional outliers not present with the other methods. The LMM and SPLMM methods both have some deviation from the normal distribution at the top end of the curve signifying that they under estimate the high BMI values. In contrast, there were an excess of extreme residual values at both ends when using the NLMM method indicating a poor fit for the data. It over estimates low BMI values and under estimates high values, thus under estimating within-individual variability and potentially leading to conservative inference about genetic associations. The male residuals displayed a similar pattern to females, although there were fewer obvious outliers. In addition, as there was less skewness in the males, the STLMM method deviated from the expected *t* distribution but in the opposite direction to that of the females, whereby the low values of BMI are underestimated. Based on model fit, all four methods were adequate in modelling childhood growth curves; however, the SPLMM was slightly better than the other methods at accounting for outliers and had the best model fit.

**Table 3:** Statistical measures used to compare model fit of the four methods.

		R^2^	R^2^ from 1,000 simulated datasets [median (IQR)]	(Observed-fitted values)^2^ [median (IQR)]	Number of SNPs detected	Average run time for genetic modelΤ (median [IQR])
Female	LMM	83.59%	83.60% (82.70, 84.44)	0.2705 (0.0579, 0.8755)	1 of 17	13.59 sec (13.41, 14.40)
	STLMM	88.78%	91.80% (86.30, 95.54)	0.2728 (0.0613, 0.9007)	3 of 17	4505 sec (4490, 4784)
	SPLMM	89.42%	89.47% (89.06, 89.84)	0.1720 (0.0374, 0.5871)	3 of 17	23.49 sec (23.41, 23.92)
	NLMM	85.98%	85.97% (85.32, 86.65)	0.1678 (0.0350, 0.5752)	2 of 51 (three tests per SNP)	0.01 sec (0.00,0.02)
Male	LMM	80.67%	80.71% (79.64, 81.71)	0.2390 (0.0470, 0.8187)	3 of 17	15.84 sec (15.66, 16.55)
	STLMM	88.72%	91.99% (87.88, 95.74)	0.2248 (0.0479, 0.8453)	4 of 17	3962 sec (3895, 3970)
	SPLMM	87.59%	87.62% (87.24, 88.03)	0.1656 (0.0329, 0.5501)	4 of 17	24.07 sec (23.78, 24.52)
	NLMM	85.10%	85.07% (84.41, 85.82)	0.1604 (0.0333, 0.5713)	5 of 51 (three tests per SNP)	0.00 sec (0.00,0.02)

Τ Median (IQR) of 100 models with the FTO SNP in R-64-bit version 2.12.1 on a 64-bit operating system with an Intel Core i7 CPU Processor (L 640 @ 2.13 GHz).

### Genetic Results

Of the 17 SNPs, a likelihood ratio test indicated the LMM method detected one significant association in the females and three in males at the 5% level of significance, the STLMM method detected three in females and four in males, the SPLMM detected three in females and four in males and finally the NLMM method detected no significant SNPs in either females or males for the size parameter but 2 significant SNPs for the velocity parameter in males. Results of all 17 SNPs can be found in Tables S2 (females) and S3 (males). The first five principal components for population stratification were not significantly associated with BMI in any of the four methods and the genetic results of the 17 SNPs remained consistent when adjusting for them (data not shown).

**Figure 2:**
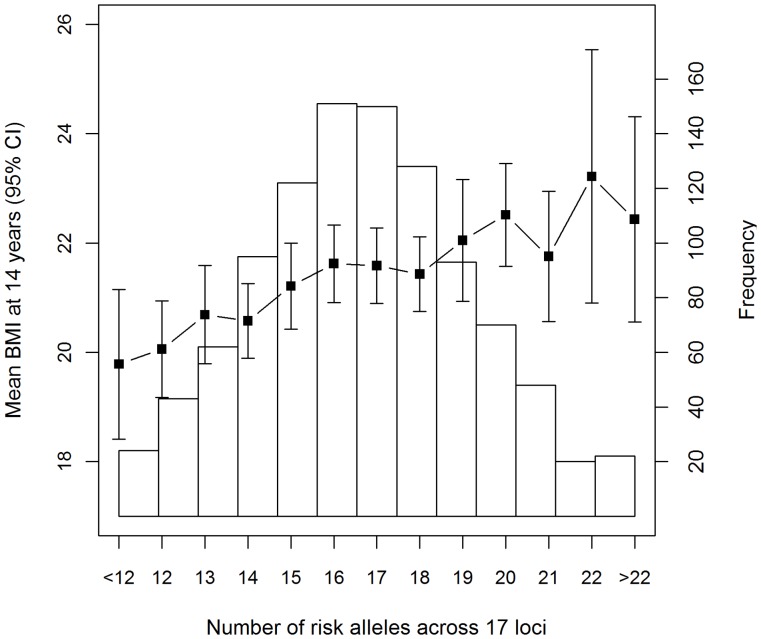
Distribution of obesity-risk allele score, with error bars for mean BMI at age 14 years. The obesity-risk-allele score incorporates genotypes from 17 loci (FTO, MC4R, TMEM18, GNPDA2, KCTD15, NEGR1, BDNF, ETV5, SEC16B, LYPLAL1, TFAP2B, MTCH2, BCDIN3D, NRXN3, SH2B1, and MRSA) in the 1,219 individuals from the Raine study with complete genetic data. The error bars display the mean (95% CI) BMI at age 14 years (the largest follow-up in adolescence) for each risk-allele score.

The obesity-risk allele score based on the genotypes at each of the 17 loci was normally distributed and showed an approximately linear association with BMI across childhood, based on the mean BMI (95% confidence interval) for each score at each age (Figure 2). When incorporating the risk-allele score into the four longitudinal models, it was associated with increasing BMI in females using all four methods however only three methods detected an association in males (Table 4). For the females, the LMM, STLMM and SPLMM methods all detected an increase in BMI per allele increase in the obesity-risk-allele-score (LMM β = 0.0046, P = 0.0216; STLMM β = 0.0492, P = 0.0410; SPLMM β = 0.0049, P = 0.0181), in addition to an increase in linear slope over time (LMM β = 0.0012, P = 0.00002; STLMM β = 0.0153, P = 0.00003; SPLMM β = 0.0012, P = 0.0006). No significant associations in the LMM, STLMM or SPLMM methods were detected for the quadratic interactions with the risk-allele score, however the cubic interaction was significant in the LMM (β = −0.00001, P = 0.0067) and STLMM (β = −0.0001, P = 0.0236). This indicates that, according the LMM and STLMM methods, females with higher allele scores plateau to adult BMI at an earlier age. In contrast, the NLMM method in both females and males was unable to detect a significant association with an increase in size or velocity, but did detect a decrease in tempo (assumed to be adiposity rebound) for each increase in risk allele. In the males, the LMM, STLMM and SPLMM methods, also detected an increase in BMI (LMM β = 0.0073, P = 0.0001; STLMM β = 0.0423, P = 0.0481; SPLMM β = 0.0071, P = 0.0001) and BMI/year per allele increase (LMM β = 0.0010, P = 0.0001; STLMM β = 0.0083, P = 0.0070; SPLMM β = 0.0008, P = 0.0068). No significant associations in the LMM, STLMM or SPLMM methods were detected for the quadratic and cubic interactions with the risk-allele score, indicating that the shape of the curve is consistent across the score categories.

**Table 4:** Results from association analysis of the obesity-risk allele score with BMI trajectory using the four methods.

		LMM			STLMM			SPLMM			NLMM			
		Beta	95% CI	P-Value	Beta	95% CI	P-Value	Beta	95% CI	P-Value		Beta	SE	P-Value
Female	Score	0.0720	0.0107, 0.1335	0.0216	0.0492	0.0020, 0.0964	0.0410	0.0758	0.0131, 0.1388	0.0181	Size	−0.0003	0.0008	0.6910
	Score*Age	0.0182	0.0099, 0.02645	1.68E-05	0.0153	0.0082, 0.0225	2.84E-05	0.0185	0.0080, 0.0290	0.0006	Tempo	−0.0090	0.0030	0.0023
	Score*Age2	−0.00001	−0.0008, 0.0008	0.9848	0.0005	−0.00004, 0.0011	0.0685	−0.0077	−0.0214, 0.0061	0.2763	Velocity	0.0045	0.0024	0.0562
	Score*Age3	−0.0002	−0.0003, −0.00004	0.0067	−0.0001	−0.0002, −0.00002	0.0236	−0.0058	−0.0128, 0.0013	0.1077				
Male	Score	0.1073	0.0553, 0.1595	0.0001	0.0423	0.0004, 0.0843	0.0481	0.1053	0.0516, 0.1591	0.0001	Size	0.0005	0.0007	0.4850
	Score*Age	0.0144	0.0074, 0.0215	0.0001	0.0083	0.0023, 0.0144	0.0070	0.0122	0.0034, 0.0210	0.0068	Tempo	−0.0072	0.0026	0.0053
	Score*Age2	−0.0006	−0.0012, 0.0001	0.1043	−0.00001	−0.0005, 0.0004	0.9586	−0.0003	−0.0120, 0.0114	0.9573	Velocity	0.0009	0.0016	0.5820
	Score*Age3	−0.0001	−0.0002, 0.000002	0.0550	−0.0001	−0.0001, 0.00003	0.1940	0.0007	−0.0052, 0.0065	0.8270				

**Figure 3:**
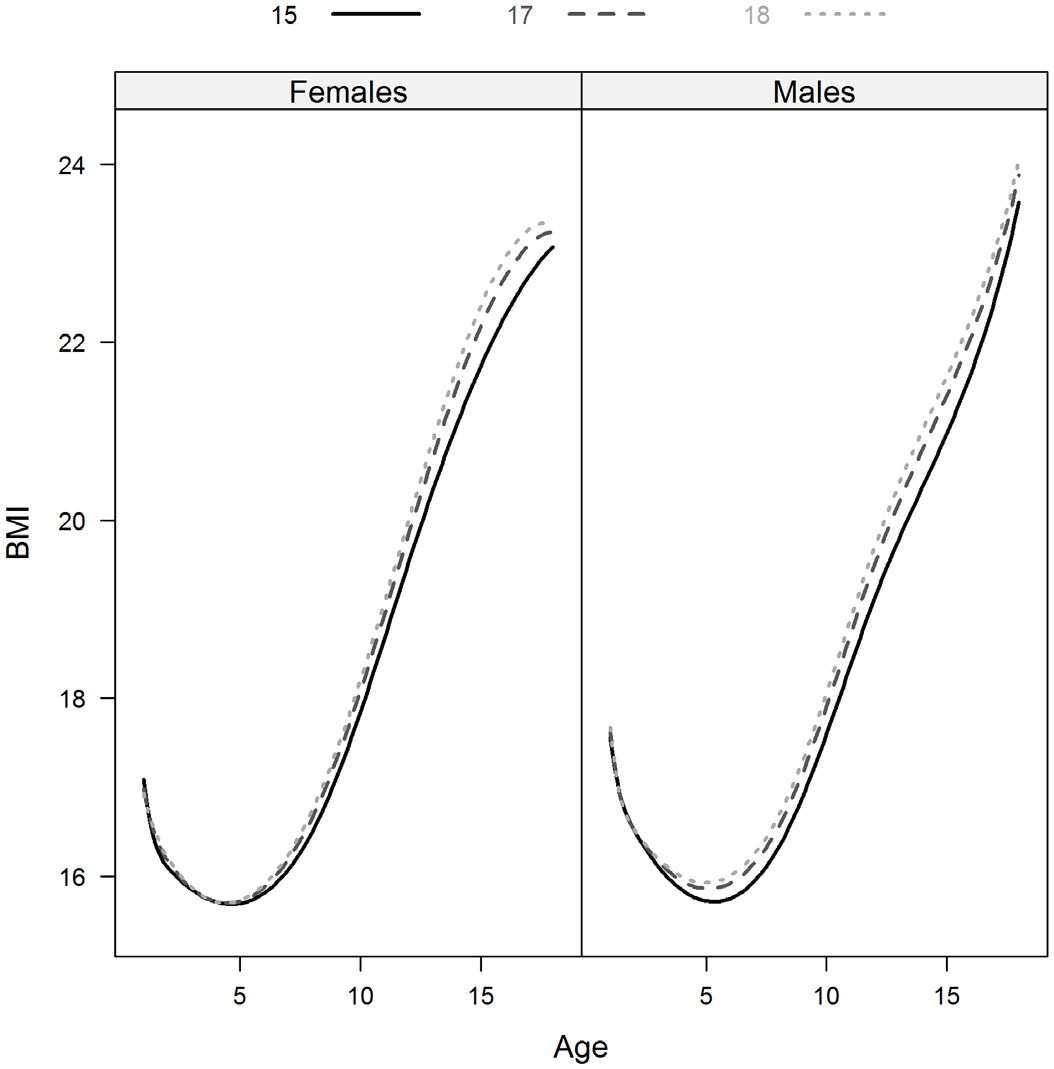
Population average curves from the SPLMM method in females and males. Predicted population average BMI trajectories from 1–18 years for individuals with 15 (lower quartile), 17 (median), and 18 (upper quartile) risk alleles in the allele score.

**Figure 4:**
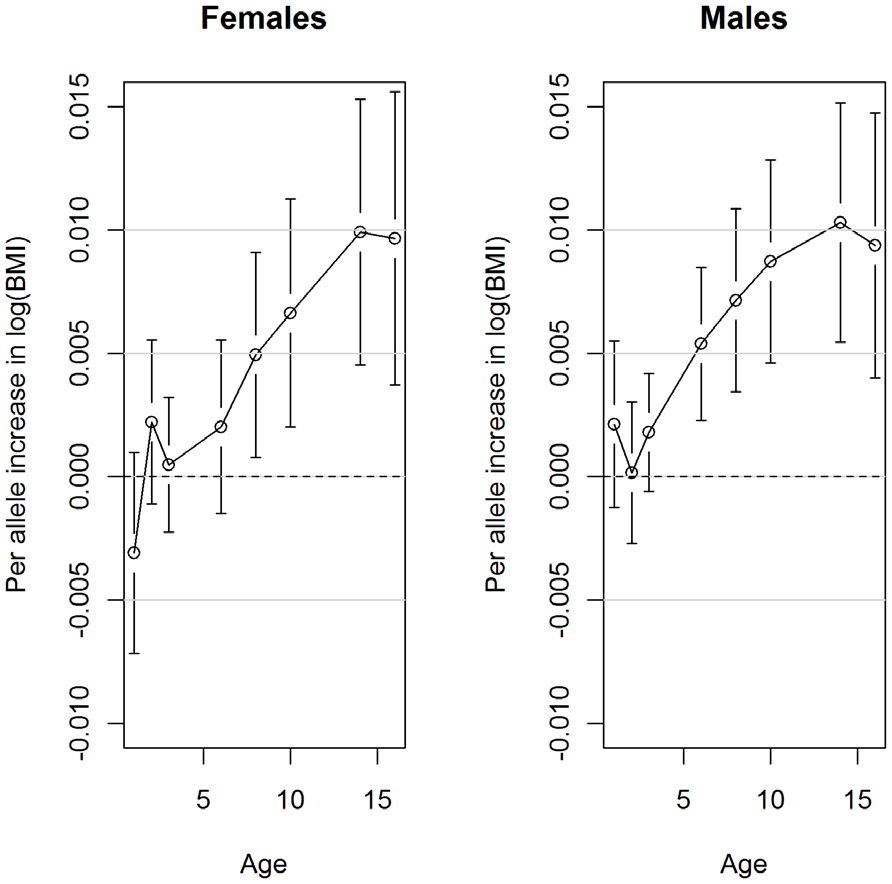
Associations between the risk-allele score and BMI at each follow-up in females and males. Regression coefficients (95% CI) presented on ln(BMI) scale from the Semi-Parametric Linear Mixed Model (SPLMM) longitudinal model, derived at each of the average ages of follow-up. For example, a male with 17 obesity-risk-alleles is likely to have an ln(BMI) 0.005 units higher at age 6 than a male with 16 risk-alleles and by age 14 this difference will be increased to 0.010 units.

Further analysis focused on the SPLMM model, as this method was shown to give the best fit to these data. There are potentially different genetic pathways leading to increased growth rate in males and females as SNPs from different genes are associated with BMI trajectory; in females, SNPs in the NRXN3, BDNF and MRSA genes were significantly associated with BMI trajectory whereas in males FTO, NRXN3, GNPDA2 and TMEM18 were significant. Figure 3 displays the population average curves for individuals with 15, 17 or 18 (25^th^, 50^th^ and 75^th^ percentile) obesity-risk alleles. The growth curves in each of the genders show different patterns; females begin their trajectory smaller than males, they have an earlier rebound, and by the age of 18 years they are beginning to plateau at their potential adult BMI. In contrast, males go through puberty at a slightly later age resulting in their BMI continuing to increase at the age of 18 years. It is apparent that the genetic effect begins later for females, around seven and a half years (P = 0.03), than males at four years (P = 0.02)(Figure 4).

## Discussion

The current study has shown that of the four statistical methods evaluated, the semi-parametric linear mixed model (SPLMM) method was the most efficient for modelling childhood growth to detect modest genetic effects in the longitudinal pregnancy cohort study investigated. In addition, we have shown that there are potentially different genetic pathways leading to increased growth rate in males and females and that the obesity-risk-allele score increases both average BMI and rate of growth throughout childhood.

There are several different statistical methods that can be used to model childhood growth. We selected four methods that would allow for adjustment of potential confounders, appropriately account for the correlation between the repeated measures, allow for incomplete data, and were computationally feasible in the context of candidate gene studies and GWAS. The evidence suggested that the SPLMM method does a better job at accounting for the variation in BMI growth than the LMM as it had a smaller residual standard deviation. The SPLMM and NLMM methods produce similar differences between observed and fitted values. The LME and STLMM methods have a larger range which indicates the prediction of BMI for each individual over time is worst using both of these methods, introducing bias whereby they over estimate low BMI values and under estimate high BMI values. As seen in the residual plots, there are a small number of outliers in this dataset, which are highly influential for both the LMM and STLMM and will effect there ability for accurate prediction. Furthermore, the estimates of skewness from the STLMM model were relatively large (intercept = 4.5791 [SE = 1.0957] and slope = 2.2336 [SE = 0.6269] for females and intercept = 2.8590 [SE = 0.5943] and slope = 1.6628 [SE = 0.4155] for males), which could be influenced by outliers and result in inaccurate predictions. Although residual plots indicate the STLMM method has the best fit to the data, it does not produce the most accurate predictions. Based on model fit, all four methods are adequate in modelling childhood growth curves; however the SPLMM produces the most accurate fitted values and can account for outliers.

Of the 17 genetic variants associated with adult BMI and obesity risk that we investigated, the SPLMM method was able to detect a higher proportion of associations with childhood growth in both males and females than the other methods. The NLMM method performed poorly in both males (five significant tests of 51) and females (two significant tests of 51) consistent with it being more conservative than the other three methods. The STLMM method detected a number of genetic effects, however it was a more computationally intensive method, which would prove difficult in larger scale genetic studies such as genome-wide association studies. Moreover, it is not as flexible as the other methods in terms of extensions to evaluate gene-environment or gene-gene interactions. The current study provides evidence that the SPLMM method is the most effective method to detect genetic associations and allows the flexibility for extensions into large scale and more complex genetic analyses.

Single genetic loci typically have small effects on complex diseases or explain only a small proportion of the variability in a quantitative trait; therefore, major increases in disease risk are expected from simultaneous exposure to multiple genetic risk variants. A *post hoc* power calculation using 1,000 non-parametric bootstrap simulations based on the Raine data indicated that this study had 97% power to detect the FTO loci rs1121980 with MAF = 0.41, which has one of the larger effect sizes on BMI, but still had 83% power to detect a more realistic smaller effect size like the BDNF SNP rs1488830 association in females with MAF = 0.21. In contrast, the power to detect the allele score, combining all risk alleles, was 95% in both males and females separately. The current study is the first to investigate, separately in males and females, an association between 17 published obesity-risk loci as an allele score and BMI trajectory throughout childhood and adolescence. Hoed et al [32] used a similar approach with a 17-loci allele-score but focused on two cross-sectional association analyses in pre−/early pubertal children and adolescents. By utilizing a longitudinal design, the current study reduced the number of genetic association tests conducted from eight in a cross-sectional setting to one per gender, reducing the necessity of adjusting for multiple testing and potentially missing important genetic loci. A second study by Elks et al [34] evaluated the association between adult obesity risk genes and growth throughout childhood using a smaller subset of obesity susceptibility loci and with analyses only up to age 11 years. Both studies conducted analysis adjusting for gender; however, this does not allow each gender to have different growth trajectories or the investigation of different timing of the genetic effects. We found substantial differences between males and females in the timing of the adiposity rebound and plateauing towards adulthood. Additionally, we detected genetic effects had different timing and effects in each gender. By combining males and females into one analysis, these genetic differences may have been averaged out and the biology underlying the differences may remain undetected.

A recent longitudinal study investigating the life-course effects of variants in the FTO gene and near the MC4R gene demonstrated that the effects strengthen throughout childhood and peak at age 20 before weakening during adulthood [33]. We detected a similar pattern with the obesity-risk allele score throughout childhood, where the effect begins around four years in males and seven years of age in females and increases in size each year. One limitation of the current study is that the cohort currently only has data available up to 18-years. It will be of interest to follow the cohort in order to investigate how the combined effect of these SNPs changes as the cohort progresses into adulthood. Further, it would be valuable to confirm that the SPLMM method is the most appropriate statistical method in other cohorts investigating the genetic determinants of childhood growth and the patterns of association across the life course.

Further studies are now required to assess the validity of these findings and also extend them to perhaps focus on interactions between genes and the environment. Interactions, both gene-gene and gene-environment, are an important area of research that is critical for understanding the mechanisms underlying obesity. We performed a small simulation study using re-sampling techniques based on 1,000 non-parametric bootstrap data sets with replacement from the Raine data and calculating the power to detect a gene-gene interaction. Two SNP combinations were investigated to gather an understanding of the range of power in our study; these included the two most commonly reported BMI associated loci, FTO rs1121980 (MAF = 0.41) by MC4R rs17782313 (MAF = 0.23) as well as two loci with large minor allele frequency, FTO rs1121980 by NEGR1 rs2815752 (MAF = 0.38). Based on these simulations, our study had 58.0% power to detect an interaction between two SNPs with larger minor allele frequencies (FTO*NEGR1) and effect sizes (FTO 0.019 kg/m^2^; NEGR1 0.011 kg/m^2^), while assuming a multiplicative model for the interaction. However, the power decreases rapidly with the minor allele frequency (FTO*MC4R) and effect size (FTO 0.0044 kg/m^2^; MC4R 0.0020 kg/m^2^) to 4.6%. We therefore believe that our study was not appropriately designed to detect gene-gene or gene-environment interactions but instead think that meta-analyses of multiple cohorts might be a better way to tackle this problem.

In conclusion, we have shown that although all four statistical methods investigated for modelling childhood growth were appropriate to model growth curves in childhood, the SPLMM method was the most efficient in these data in terms of predicted values and detection of genetic effects. Further, we have shown that there is some evidence that genetic variations in established adult obesity-associated genes are associated with childhood growth; however these effects differ by gender and timing of effect. This study provides further evidence of genetic effects that may identify individuals early in life that are more likely to rapidly increase their BMI through childhood, which provides some insight into the biology of childhood growth.

## Supporting Information

Table S1 **Details of the 17 SNPs used in genetic association analyses.**
(XLSX)

Table S2 **Results of genetic association analysis in females for all 17 SNPs in each of the four statistical methods.**
(XLSX)

Table S3 **Results of genetic association analysis in males for all 17 SNPs in each of the four statistical methods.**
(XLSX)
